# Children’s daily activities and knowledge acquisition: A case study among the Baka from southeastern Cameroon

**DOI:** 10.1186/s13002-015-0072-9

**Published:** 2015-12-24

**Authors:** Sandrine Gallois, Romain Duda, Barry Hewlett, Victoria Reyes-García

**Affiliations:** Institut de Ciència i Tecnologia Ambientals, Universitat Autònoma de Barcelona, 08193 Bellaterra, Spain; Museum national d’Histoire naturelle, Paris, France; Department of Anthropology, Washington State University, Vancouver, WA USA; Institució Catalana de Recerca i Estudis Avançats (ICREA), Barcelona, Spain

**Keywords:** Cultural transmission, Embodied knowledge, Ethnoecology, Hunter-Gatherers, Learning

## Abstract

**Background:**

The acquisition of local knowledge occurs through complex interactions between individual and contextual characteristics: as context changes, so it changes the acquisition of knowledge. Contemporary small-scale societies facing rapid social-ecological change provide a unique opportunity to study the relation between social-ecological changes and the process of acquisition of local knowledge. In this work, we study children’s involvement in subsistence related activities (i.e., hunting and gathering) in a context of social-ecological change and discuss how such involvement might condition the acquisition of local knowledge during childhood.

**Methods:**

We interviewed 98 children from a hunter-gatherer society, the Baka, living in two different villages in southeastern Cameroon and assessed their involvement in daily activities. Using interviews, we collected self-reported data on the main activities performed during the previous 24 h. We describe the frequency of occurrence of daily activities during middle childhood and adolescence and explore the variation in occurrence according to the sex, the age group, and the village of residency of the child. We also explore variation according to the season in which the activity is conducted and to the predicted potential of the activity for the acquisition of local knowledge.

**Results:**

Baka children and adolescents engage in subsistence-related activities (i.e., hunting and gathering) and playing more frequently than in other activities (i.e., traditional tales or schooling). Gender differences in children’s subsistence activities emerge at an early age. Engagement in activities also varies with age, with adolescents spending more time in agricultural activities, modern leisure (i.e., going to bars), and socializing than younger children. When conducting similar activities, adolescents use more complex techniques than younger children.

**Conclusion:**

Subsistence activities, which present a high potential for transmission of local knowledge, continue to be predominant in Baka childhood. However, Baka children also engage in other, non-traditional activities, such as modern forms of leisure, or schooling, with a low potential for the transmission of local knowledge. Baka children’s involvement in non-traditional activities might have unforeseen impacts on the acquisition of local knowledge.

## Background

“*It is not of no little importance what sort of habits we form from an early age - it makes a vast difference, or rather all the difference in the world*” (Aristotle in Ochs & Izquierdo [1]).

The acquisition of local knowledge occurs through complex interactions between the individual and her contextual characteristics [2, 3]. Among these contextual characteristics, daily life experiences are essential determinants in the acquisition of cultural knowledge as they shape not only the kind of knowledge being learned, but also the way such knowledge would be learned along the lifespan [4–6]. In this sense, scholars have argued that the acquisition of knowledge occurs through a process of embodiment, or enskillment, which is directly linked with the practical engagement of the individuals in their surrounding environment [7, 8], including both the physical and the social settings [4, 5]. Furthermore, four decades ago, John and Beatrice Whiting [9] proposed that cultural aspects, and specifically local maintenance systems, shape where children go and with whom (i.e., their physical and social setting) and -therefore- what and how they learn. In other words, the way in which children spend their time is largely dependent upon the needs of parents to do particular subsistence tasks, and children learn from these culturally established settings [10]. An empirical validation of such ideas, focusing on the discussion of how cultural setting affects the acquisition of local knowledge, can be found in recent work by Boyette [11] with the Ngandu and the Aka in Central African Republic. In this work, he shows how the knowledge of children living in the same environment but from two different societies with different subsistence patterns largely differs.

Given that the acquisition of knowledge occurs through complex interactions between various factors which occur “*both within and without the individual, and of the spatial and temporal arrangements in which these interact*” [12] (pg.S5) and in which both the mind and the body are involved [3], the analysis of context is of great importance for understanding the acquisition of knowledge. With such premise, the question we ask here is: how the process of acquisition of knowledge changes as context change? If the process of knowledge acquisition is largely dependent on context, in situations of rapid change such process might be largely affected.

In this work, we address the question by analyzing involvement in daily activities among children from a contemporary small-scale society facing rapid social-ecological change. Specifically, we explore the variation in activity occurrence according to the sex, age category, season, and village of residency of the child. Then, we discuss how such involvement might condition the acquisition of local ecological knowledge (LEK), or the knowledge, practices and beliefs related to the environment, during childhood. The Baka hunter-gatherers from the Congo Basin represent an ideal case to study such problematic as they have faced several drastic social-ecological changes since the middle of the last century, with resulting changes in their livelihood. We focus on children because several scholars have reported that -in small-scale societies- most cultural knowledge is acquired before adolescence [13, 14], sometimes even before 10 years of age [15], thus suggesting that childhood is a key period for the cultural knowledge acquisition. We focus on children’s involvement on daily activities as such behavior might provide insights into the nature of knowledge acquisition. Given the trade-offs in the acquisition of different types of knowledge [16, 17], the involvement into particular activities might help understand how the process of knowledge acquisition operates. Moreover, the way in which children invest their time might be critical to understand preferences for the acquisition of different types of knowledge [17, 18] and can therefore potentially help predict changes in the transmission of different bodies of knowledge. Additionally, we chose to emphasize local ecological knowledge, as such knowledge systems are an essential component of human societies, especially for the subsistence and the wellbeing of hunter-gatherer societies [19].

## The Baka

The Baka are one of several hunter-gatherer groups living in the tropical forest of the Congo Basin. Their population, estimated at around 30.000 people, spreads across four countries: most Baka live in Cameroon, but some groups are found in the Democratic Republic of Congo, Gabon and Central African Republic. The Baka have been extensively studied (see for example [20–23]), so, rather than providing a complete ethnographic description, in this section we provide a brief general overview of the Baka and then focus on describing Baka childhood.

Until recently, the Baka were highly nomadic, moving between several forest camps and living on forest resources and on the exchange of products with Bantu speaking neighboring farmers. However, over the last 50 years, the Baka have experienced several important social changes. First, new outsiders, including missionaries, poachers, logging and mining companies, and members of international organizations representing conservationists’ interests, have arrived to the territory occupied by the Baka. While interests vary from actor to actor, for the Baka, their arrival to the area has resulted in a gradual reduction in access to forest resources in general and to game and wild edibles in particular. Second, as a result of their reduced access to forest resources, Baka began to leave their forest camps, a shift reinforced by the influence of missionaries and government promoted sedentarization programs which, since the 1950’s, led many Baka to establish themselves in settled villages [20, 23–25]. Consequently, today most Baka settlements are found along logging roads, some of them in proximity to Bantu speaking villages. Moreover, many Baka have started to engage in agricultural work, both by opening their own plots and by providing casual labor to neighboring Bantu villagers [23]. A third important change relates to the increase of school attendance, facilitated by sedentarization. Schooling was first made available to Baka children by the missionaries and non-governmental organizations. As a result of all these changes, many Baka nowadays have adopted a mixed forager-horticulturalist subsistence strategy.

Differently from Western views of childhood, but similar to how childhood has been described in other small-scale societies [26, 27], Baka children are highly autonomous from an early age. In a way, Baka children receive the same treatment than adults, even if it is understood that they are in a learning process. For example, Baka children have the freedom to make their own decisions, but they are also considered responsible for the consequences of such decisions. Baka children are also expected to participate on daily household chores such as fetching water, bringing meals to neighboring households, or collecting firewood. However, very few obligations are imposed upon Baka children, and physical punishment is rare. Another important element during Baka childhood is allo-parental care, or the acting as parents of individuals other than the parents. Thus, it is common that older siblings, grand-parents or other adults take care of a child [28, 29]. Moreover, among the Baka, it is assumed that older sisters are the secondary caregivers of infants [30]. Since Baka mothers restart productive activities soon after giving birth, but since Baka infants are mostly held, it is common that children-specially girls- as young as 6 years of age are asked to accompany their mother to help taking care of infants. Due to the importance of allo-parental care, Baka children witness a high degree of physical and emotional intimacy with others, including older siblings but also adults outside their nuclear family [27].

## Methods

Our study took place in several Baka communities of the department Haut-Nyong, in southeastern Cameroon, where we collected qualitative and quantitative data during 18 months, from February 2012 to May 2014. We obtained free prior and informed consent in all the villages from every individual participating in this study, as well as the consent of all the parents of the children we worked with. This study adheres to the Code of Ethics of the International Society of Ethnobiology and has received the approval of the ethics committee of the Universitat Autònoma de Barcelona (CEEAH-04102010).

### The sample

Intensive field work was conducted in two communities, comprising 264 and 410 individuals, of which 145 and 208 were children (or people <16 years of age). Both communities differed in their proximity to the village of Bantu-speaking neighbors- the Nzime- and in the type of school. The first village is settled on the prolongation of the Nzime farmer’s village and Baka children have the opportunity to attend a public national school, together with Nzime children. In contrast, the second village is located at approximately 2 km from closest Nzime neighbor village and has a private school managed by a local institution promoting schooling among the Baka.

The sample for this research included all the children between 5 and 16 years of age willing to participate. The upper limit was fixed at 16 years of age because at this age, the Baka generally start a separate household and are thus considered adults. Although evidence exists that the transmission of local knowledge starts earlier [31], the lower limit was fixed at 5 years of age because younger children were generally too shy or too unreliable to answer interview questions.

### Data collection

Over the whole period of data collection, the two first authors lived in the two selected Baka communities, following Baka socio-cultural norms and participating on the daily life of neighboring households, e.g., while washing clothes, cooking, taking care of children, accompanying them on fishing, hunting and gathering expeditions and to their forest camps and agricultural plots. Participant observation was conducted among adults and children and with as many households as possible.

#### Qualitative data collection

During the first 6 months of fieldwork, we observed children’s daily life. Additionally, we conducted spontaneous discussions and semi-structured interviews with adults and children about children’s daily activities. Initially, most interviews were performed with the company of a translator. Eventually, we learned enough Baka to be able to communicate directly with informants. To get a better understanding of children’s daily life, we followed groups of boys and girls of different ages in their daily activities. During such observations, we noted the composition of the group of individuals, the location of the activity, and the total time invested in the activities performed. We beware of following groups of girls and boys of different ages. The qualitative information collected with such method provided us an overview of Baka livelihoods and of the main patterns of children’s activities. Moreover, in addition to being at the basis of our questionnaire design, information collected with qualitative methods has been largely used in the discussion section to interpret our findings.

#### Quantitative data collection

Quantitative data collection methods included a census of all the individuals living in both studied villages and a questionnaire on children’s daily activities. The census included the name, age, clan, kinship data, and level of education of all children in the sample. As most Baka cannot recall their date of birth nor have birth records, we used kinship information (i.e., order of birth) to estimate the age of children in our sample.

To assess children’s involvement in daily activities, we used a systematic interview protocol consisting on asking children whether they had performed a set of selected activities during the 24 h previous to the interview. First, we established a list of the activities most frequently conducted by children. The list was constructed using etic and emic inputs: we used information from semi-structured interviews and participant observation to identify the activities performed by boys and girls between 5 and 16 years of age. We then grouped these activities into 15 clusters of similar activities. For example, we clustered together different types of hunting or fishing with different techniques. Then, during the systematic interview, we asked children to report all the activities they had performed since the previous day at the time of the interview and coded the activities listed in one of the 15 clusters of activities. After children had finished spontaneously listing activities, we asked whether they had also performed any of the other activities pre-defined in our list. We conducted a total of 232 interviews with 102 children, 53 boys and 49 girls, which represents 34 and 64 % of the children in the selected range age in the studied villages.

### Data analysis

As the main goal of this work is to discuss children’s involvement in daily activities in relation both to the process of LEK acquisition, we grouped the 15 categories of activities in three different clusters: a) subsistence-related activities that may favor the acquisition of LEK; b) activities indirectly-related to subsistence but that might favor the acquisition of LEK through norms, values and cosmology; and c) activities recently introduced in Baka livelihoods, which are unlikely to favor the acquisition of LEK. We are aware that the notion of subsistence is disputed [32], but for the purpose of this work we included in the category of subsistence-related activities those activities that procure essential elements for living, including both nutrition and shelter. We also included activities related to the procurement and processing of resources. Consequently, our first cluster includes household maintenance, hunting, gathering, agricultural work, fishing and handicraft and considers both work-playing and actual work. Our second cluster includes traditional singing and dancing, storytelling, maintenance, and playing, but only when the games were not related to subsistence activities from the previous cluster. Finally, our third cluster includes activities which have been mostly introduced more or less recently, i.e., in the last five decades. Such cluster includes activities like attending school, playing football, listening to music, alcohol drinking and socializing with Nzime people (see Table 1).

**Table 1:** Description of children’s activities

Cluster	Category	Activities
Subsistence-related activities	Household maintenance	Fetching water; collecting firewood; washing clothes/dishes; sweeping house; cooking.
	Hunting	Hunting small animals with traps; hunting with sling, bow and arrows, or stones; collective hunting; hunting cable snares; hunting with spear; unearth game with smoke; wheel and “*lékà*” playing.
	Gathering	Gathering of sub-spontaneous and/or wild edibles products.
	Agricultural Work	Cleaning fields; planting or harvesting agricultural products in household’s or Nzime’s plots
	Fishing	Collective fishing with dams; fishing with hook; fishing with net.
	Handicraft	Making toys in *Raphia sp.*; building replicates of ; weaving mats and baskets.
Non-subsistence related activities	Play^a^	Play with Baka children; collective plays, such as hide-and-seek, and marbles; solitary plays as wheel/car pushing.
	Maintenance	Sleeping; resting; eating with Baka’.
	Traditional songs, tales and dances	Performing Baka’s songs and dances; narrating tales; listening to tales.
Recently introduced activities	Listening to music	Listening to modern music
	School	Attending school; doing homework
	Other activities	Trade, hairdressing…
	Football	Football playing
	Alcohol drinking	
	Socializing with Nzime children	Spending time with Nzime children (play, chatting…)

^a^This category only includes leisure play not related to subsistence activities; plays related to subsistence or work-playing are part of the first cluster

It is worth highlighting that the performance of any of the listed activities might contribute to the acquisition of local ecological knowledge. Indeed, as all the activities a child performs are embedded in the Baka’s culture and environment, they can potentially contribute to local ecological knowledge acquisition. However, given the content of the different activities listed, we assume that some activities are more likely to improve the acquisition of LEK than others. For example, activities directly related to subsistence, in addition to be developed in Baka social and environmental context, require the child to use her skills and put her knowledge into practice. This largely contrasts with other activities, such as listening to modern music, where the child would be less likely to involve ecological knowledge and skills necessary to subsistence activities.

We aggregated data on children’s daily activities by computing the frequency with which each of the 15 categories of activities was reported by each child. To avoid the potential biases derived from the overrepresentation of children who were interviewed more than others, we randomly selected one observation per child (*n* = 102). Frequency was coded as 1 (if the activity was performed) or 0 (otherwise), independently of whether the activity was performed more than once during the 24-h recall (i.e., if a child reported hunting with traps and hunting with bows and arrows we only coded hunting as 1).

Using census data, we analyzed Baka children’s engagement in different activities by sex and age. Age categories were determined drawing on bibliographic references [33, 34] and our own interviews with Baka adults and consisted in i) middle childhood (>5–9 = <years-old), ii) pre-adolescence (<9–13 = < years-old), and iii) adolescence (<13–16 > years-old). We also compared involvement in the different activities from children in the two studied villages. Then, since our data were collected during two different seasons - the major dry season (from beginning of February to mid March) and the minor rainy season (from mid March to the end of June) - we also analyzed seasonal variation.

To assess differences in children’s involvement in daily activities by sex, village and season we calculated the difference of proportions [35]. For that aim, we first calculated the relative proportion of each cluster of activities (i.e., the number of observations reported by children in each cluster of activities from the total amount of observations reported) and for each of the groups of analysis (i.e., girls/boys, village closest/furthest to neighbor’s village, and major dry/minor rainy season). We then ran two-sample test of proportions at the level of 95 % of confidence, to evaluate whether spotted differences were statistically significant [35]. We tested for significant differences across age categories by running Fisher’s exact tests for each cluster of activities. In a final analysis, we used multivariate logistic regression models to analyze the association between sex, age category, seasonality, and village of residency with each of the 15 types of activities.

## Results

### Children’s main daily activities

The cluster of subsistence-related activities emerges as the one with most commonly performed activities. Moreover, several subsistence-related activities are performed every day by about half of the sample. Activities indirectly related to subsistence and recently introduced activities were less common (Table 2).

**Table 2:** Children’s involvement in daily activities. For the full sample and by sex

Cluster	Category	Full sample		Girls		Boys		Results of two-proportion tests
		Frequency (*n* = 102)		Frequency (*n* = 49)		Frequency (*n* = 53)		
		*N*	%	*N*	%	*N*	%	Z
Subsistence-related activities	Household maintenance	97	95.1	49	100	48	90.6	2.2**
	Gathering	50	49.0	28	57.1	22	41.5	1.57
	Hunting	46	45.1	9	18.4	37	69.8	−5.21***
	Agricultural work	32	31.4	18	36.7	14	26.4	1.12
	Fishing	26	25.5	17	34.7	9	17	2.05**
	Handicraft	12	11.8	3	6.1	9	17	−1.71*
Non-subsistence related activities	Plays	60	58.8	29	59.2	31	58.5	0.07
	Maintenance	25	24.5	9	18.4	16	30.2	−1.38
	Traditional songs, tales and dances	9	8.8	6	12.2	3	5.7	1.16
Recently introduced activities	Listening to music	46	45.1	25	51	21	39.6	1.16
	School	22	21.6	11	22.4	11	20.8	0.2
	Other activities	13	12.7	4	8.2	9	17	−1.33
	Football	13	12.7	1	2	12	22.6	−3.12***
	Alcohol drinking	12	11.8	5	10.2	7	13.2	−0.47
	Socializing	6	5.9	1	2	5	9.4	−1.59

*p > .1; **p < .05; ***p < .01

Among the subsistence related activities, household maintenance was predominant. Children mentioned having conducted household maintenance in almost 95 % of the interviews. Other common activities in this cluster included hunting and gathering, reported in 45 and 49 % of the interviews. Agricultural work was reported in 31 % of the interviews, fishing in almost 26 %, and handicraft in 12 %.

Among the non-subsistence related activities, playing appears as the most frequently mentioned activity, reported in almost 59 % of the interviews. Overall, playing is the second most frequently cited activity, after household maintenance.

Finally, among the recently introduced activities, listening to music was reported in 45 % of the interviews, whereas attending school was only reported in 22 % of the interviews.

### Gendered involvement in daily activities

Boys and girls show different levels of involvement in most, but not all, activities (Table 2). Although household maintenance is very common for both, we still found statistically significant differences between boys (almost 91 % of the interviews) and girls (100 % of the interviews). A more detailed analysis suggests that there are also important differences in the specific activities performed (Table 3). Thus, fetching water was listed in 71 % of the interviews conducted with girls, but only in 58 % of the interviews conducted with boys; similarly cooking was mentioned in 92 % of the interviews conducted with girls, but only in 13 % of the interviews conducted with boys.

**Table 3:** Frequency and percentage of listing activities related to household maintenance, by sex

Activities	Girls		Boys	
	*N*	%	*N*	%
Cooking	45	91.8	23	13.4
Caretaking Children/Babies	17	34.7	11	20.8
Fetching Water	35	71.4	31	58.5
Collecting Firewood	13	26.5	10	18.9
Washing Clothes/Dishes	16	32.7	11	20.8

Statistically significant differences also exist in the frequency with which boys and girls perform hunting and fishing activities. Hunting appeared as a male orientated activity, with 70 % of the boys but only 18 % of the girls reporting hunting (Table 3). In contrast, fishing is a girl-oriented activity, with girls reporting fishing twice as often as boys (35 vs.15 %). In the same line, but to a lower extent, gathering tends to be more a girl- than boy-oriented activity (cited in 57 % of the interviews with girls and in 41 % of the interviews with boys). From all the activities in the cluster of subsistence-related activities, the only one that seems to be reported with similar frequency between boys and girls is agricultural work.

Girls and boys seem to engage with the same frequency in activities indirectly related to subsistence, with one exception: girls report performing traditional songs, tales, and dances more frequently than boys (12 vs. 6 %; Table 2). Regarding recently introduced activities, the only statistically significant difference between girls and boys relates to playing football, a male-oriented activity in the study area (23 % for boys vs. 2 % for girls) (Table 3).

### Variation among age-sex categories

**Fig. 1:**
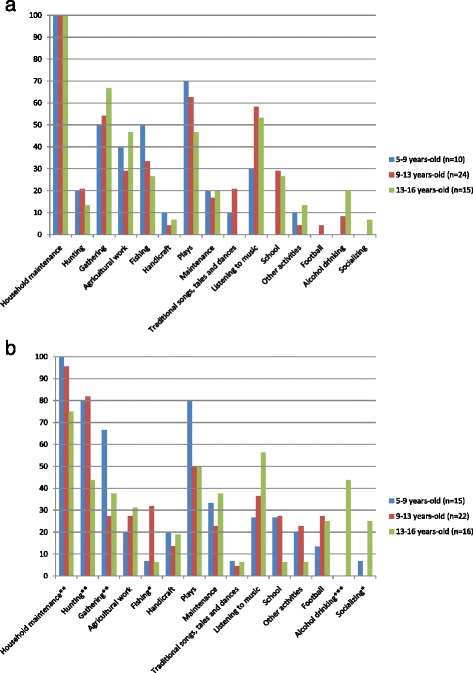
Percentage of children’s activities by sex and age category. **a** among girls; **b**: among boys); *p < .1; **p < .05; ***p < .01 to the Fisher’s exact tests

Given the magnitude of the gendered differences found, we kept the sample of boys and girls separated to analyze children’s involvement in daily activities by age-categories (Fig. 1a). Overall, the analysis shows differences in time investment as girls and boys move from middle childhood to adolescence. Among the activities related to subsistence, both girls and boys perform more hunting activities but play less frequently as they grow up. In contrast, they spend more time performing non-traditional activities, such as socializing, listening to music and drinking alcohol. As girls move into adolescence, they invest less time in fishing, (27 % of adolescent girls’ interviews vs. 50 % of middle childhood girls) (Fig. 1). In the same line, adolescent girls invest even less time in hunting than middle childhood girls do (13 vs. 20 %). Contrarily adolescent girls are more frequently involved in agriculture (47 vs. 40 %) and in gathering (67 vs. 50 %) than middle childhood girls.

Although the differences on the frequencies of activities between age categories appear higher among girls, we only found statistically significant differences in the sample of boys (Fig. 1b). Thus, as boys enter adolescence, they become less frequently involved in hunting (reported in 44 % of the interviews with adolescent boys vs. 80 % of the interviews with middle childhood boys). Differently to girls, as they grow up, boys are less frequently involved in household maintenance activities. Also in contrast to the pattern found among girls, adolescent boys allocate less time than younger boys to gathering (38 % for adolescents vs. 67 % for middle childhood boys).

Differences also appeared when comparing the activities of children from different age categories (Table 4). Overall, relatively easy tasks, like fetching water, firewood collecting, or gathering easy target edibles are mostly performed by children from middle childhood. Such practices tend to become more complex as children grow up. For instance, it is only after reaching adolescence that the Baka start using spears, setting cable snares, gathering honey, and processing edibles, such as palm wine and fruit’s oil. It is also at this age that they start opening and managing their own plot.

**Table 4:** Variation in the performance of specific tasks by children during their subsistence activities, by age category of the children

Category of activities	Specific task	5–9 years-old		9–13 years-old		13–16 years-old	
		Boys	Girls	Boys	Girls	Boys	Girls
Hunting	Putting vegetal snare for mice	X		X		X	
	Putting vegetal snare for birds	X		X		X	
	Putting snares with iron cables					X	
	Hunting small game with sling	X		X		X	
	Hunting small game with bow and arrows	X		X		X	
	Playing “*leka*”	X	X	X	X	X	X
	Playing wheel			X		X	
	Small game collective hunting	X	X	X	X	X	X
	Unearthing game with smoke			X	X	X	X
	Hunting with spear			X		X	
Gathering	Sub-spontaneous tubers gathering	X	X	X	X	X	X
	Palm seed processing	X	X	X	X	X	X
	Palm seed gathering			X	X	X	X
	Mushroom gathering	X	X	X	X	X	X
	*Gnetum*’s leaves gathering				X		X
	Climb to gather papaya/avocat’s fruits	X	X	X	X	X	X
	Honey gathering					X	
	Palm wine gathering and processing					X	
Agriculture	Weeding	X	X	X	X	X	X
	Planting	X	X	X	X	X	X
	Harvesting Groundnuts	X	X	X	X	X	X
Fishing	Fish gathering	X	X	X	X	X	X
	Fishing with hook	X		X		X	
	Building dam			X	X	X	X
	Water extracting	X	X	X	X	X	X
	Dig the river			X	X	X	X

### Differences between villages

Overall, we found few differences between the two studied villages: the only statistically significant differences appear in agricultural activities, school, and alcohol drinking (Table 5). Children in the most isolated village, reported engaging in agriculture two-times more often than children from the other village (almost 43 vs. 16 %). Children reported drinking alcohol more frequently in the village further to Nzime’s village than in the other village (19 vs. 2 %). In the same village, where the school is run by missionaries, children also attend school more frequently than in the village closer to the Nzime village, where the school is shared with the Nzime (almost 28 vs. 14 %).

**Table 5:** Children’s involvement in daily activities, by village

Cluster	Category	Village closer to Nzime’s village Frequency (*n* = 44)		Village further to Nzime’s village Frequency (*n* = 58)	Results of two-proportion tests	
		*N*	%	*N*	%	Z
Subsistence-related activities	Household maintenance	43	97.7	54	93.1	−1.07
	Hunting	16	36.4	30	51.7	1.54
	Gathering	20	45.5	30	51.7	0.63
	Agricultural Work	7	15.9	25	43.1	2.93***
	Fishing	14	31.8	12	20.7	−1.28
	Handicraft	4	9.1	8	13.8	0.73
Non-subsistence related activities	Plays	23	52.3	37	63.8	1.17
	Maintenance	7	15.9	18	31	1.76*
	Traditional songs. tales and dances	3	6.8	6	10.3	0.62
Recently introduced activities	Listening to music	17	38.6	29	50	1.14
	School	6	13.6	16	27.6	1.7*
	Other activities	8	18.2	5	8.6	−1.43
	Football	6	13.6	7	12.1	−0.24
	Alcohol drinking	1	2.3	11	19	2.59**
	Socializing	4	9.1	2	3.4	−1.2

* *p* > .1; **p < .05; ***p < .01

### Seasonal variation

Children’s involvement in different activities does not seem to largely vary between the two seasons when data were collected (Table 6). Nevertheless, we found statistically significant differences for two subsistence activities that were more frequently performed during the dry than during the minor rainy season: hunting and agriculture. Hunting was mentioned in 63 % of the interviews conducted during the dry but only in 33 % of the interviews conducted during the minor rainy season. Likewise, agricultural work was listed in 44 % of the interviews conducted during the dry but only in 23 % of the interviews conducted during the rainy season. To a smaller extent, gathering and fishing were also more frequently performed during the dry season (54 vs. 46 % for gathering and 29 vs. 23 % for fishing).

**Table 6:** Children’s involvement in daily activities, by season (18 girls and 23 boys during dry season; and 31 girls and 30 boys during the rainy season)

Cluster	Category	Dry Season		Rainy season		Results of two-proportion tests
		Frequency (*n* = 41)		Frequency (*n* = 61)		
		*N*	%	*N*	%	Z
Subsistence-related activities	Household maintenance	38	92.7	59	96.7	−0.93
	Hunting	26	63.4	20	32.8	3.05***
	Gathering	22	53.7	28	45.9	0.77
	Agricultural Work	18	43.9	14	23	2.24**
	Fishing	12	29.3	14	23	0.72
	Handicraft	5	12.2	7	11.5	0.11
Non-subsistence related activities	Plays	22	53.7	38	62.3	−0.87
	Maintenance	13	31.7	12	19.7	1.39
	Traditional songs. tales and dances	3	7.3	6	9.8	−0.44
Recently introduced activities	Listening to music	18	43.9	28	45.9	−0.2
	School	9	22	13	21.3	0.08
	Other activities	3	7.3	10	16.4	−1.37
	Football	5	12.2	8	13.1	−0.14
	Alcohol drinking	8	19.5	4	6.6	1.99**
	Socializing	2	4.9	4	6.6	−0.35

*p > .1; **p < .05; ***p < .01

Some activities indirectly related to subsistence show seasonal differences. Playing was reported more frequently during the minor rainy than during the dry season (62 vs. 54 %), a seasonal distribution that probably relates to the higher frequency of subsistence activities conducted during the dry season. Interestingly, children also seem to perform more frequently maintenance activities during the dry than during the minor rainy season (32 vs. 20 %). Finally, whereas children tend to perform recently introduced activities with the same frequency during both seasons, alcohol drinking is almost three-fold more frequent during the dry than during the rainy season (20 vs. 7 %).

### Correlates of children’s involvement in daily activities

Logistic regression analysis mostly confirms results from bivariate analysis (Table 7). Namely, a set of multiple logistic regressions with the frequency of performance in the different categories as dependent variables show that differences between both sexes are statistically significant for most activities included in the cluster of subsistence related activities. Hunting is a boy-oriented activity (odd ratio = 13.6. *p* < 0.001), whereas gathering (odd ratio = 0.5. *p* > 0.1) and fishing (odd ratio = 0.37. *p* < 0.05) are girl-oriented activities. Since all girls performed household maintenance, we cannot compute a coefficient for this activity. Boys are much more frequently involved in football than girls (odd ratio = 14.75. *p* < 0.05). The variable sex is not statistically significant for the other activities.

**Table 7:** Results of multivariate logistic regression among the different activities

Cluster	Category	Pseudo R^2^	Predictors			
			Male	Age	Dry Season	Village closer to Nzime’s village
Subsistence-related activities	Household maintenance	0.17	omitted	0.72*	1.56	1.77
	Hunting	0.30	13.6***	0.86*	0.20***	0.87
	Gathering	0.03	0.50*	0.95	0.76	0.79
	Agricultural Work	0.11	0.53	1.11	0.56	0.32*
	Fishing	0.08	0.37**	0.94	0.44	2.37
	Handicraft	0.05	3.09	1.01	1.22	0.62
Non-subsistence related activities	Plays	0.08	0.94	0.83***	2.34*	0.35**
	Maintenance	0.05	1.83	0.99	0.72	0.50
	Trad. songs, tales and dances	0.04	0.41	0.95	1.8	0.45*
Activities not involving LEK acquisition	Listening to music	0.05	0.62	1.13*	1.36	0.57
	School	0.04	0.84	0.95	1.46	0.34*
	Other activities	0.07	2.60	0.98	2.04	1.95
	Football	0.15	14.75**	1.06	1.12	1.34
	Alcohol drinking	0.45	1.27	2.06***	0.75	0.15
	Socializing	0.26	6.07	1.63**	0.43	11.14

Cells include the odd ration. *p < .10; **p < .05; ***p < .01

Multivariate models also confirm the importance of age in explaining children’s involvement in daily activities. Thus, older children engage less frequently in household maintenance (odd ratio = 0.72. *p* < 0.1) and hunting (odd ratio = 0.86. *p* < 0.1). Age is also negatively associated to play (odd ratio = 0.83. *p* < 0.01), but positively associated to alcohol drinking (odd ratio = 2.06. *p* < 0.01), socializing (odd ratio = 1.63. *p* < 0.5) and, to a lower extent, listening to music (odd ratio = 1.13. *p* < 0.1).

Finally, multivariate regressions also show that there are some differences between both villages but not between seasons. Thus, children from the village closest to Nzime’s village tend to be less engaged in agricultural work (odd ratio = 0.32. *p* < 0.1), play (odd ratio = 0.35. *p* < 0.5), traditional songs and dances (odd ratio = 0.45. *p* < 0.1), and school attendance (odd ratio = 0.34. *p* < 0.1) than children from the other village. In relation to the season, the only statistically significant associations found were a lower frequency of hunting during the dry season (odd ratio = 0.2. *p* <0.01) and a higher frequency of play during the dry season (odd ratio = 2.34. *p* < 0.1).

It is worth noting that most multiple logistic regressions explain a relatively small fraction of the variation found (between 3 and 45 %), which suggest that other variables not accounted for in our model do affect children’s involvement in daily activities. The two models with higher predictive power are the model for hunting, which explained almost 30 % of the variation found, and the model for alcohol drinking (45 %).

## Discussion

We organize the discussion around two main results derived from our analysis: a) differences in children’s involvement in daily activities according to sex and age-category, and b) the relative importance of different activities in relation to their potential for LEK acquisition.

### Variations in Baka childhood activities

A main finding of our work is that, irrespectively of their sex and age category, most Baka children engage in household maintenance, a finding that has also been reported among other small-scale societies [11, 13]. Thus, from the earliest age, Baka children are expected to participate in household chores, for example helping their mothers with tasks such as collecting firewood, fetching water, and taking care of younger children. But, differently to what has been reported in farmer societies [36], Baka children are not expected to participate in income generating activities (although some of them do, especially after reaching adolescence). While such situation that has led some researchers to coin the notion of ‘children in paradise’ [37], Baka children do frequently engage in productive subsistence activities (i.e., hunting, gathering, fishing, and agricultural labor). Our ethnographic observations suggest that Baka children perform these subsistence activities mostly out of enjoyment, especially as these activities are often embedded in games [38]. However, it should also be noticed that such activities seem to provide an important part of children nutritional intake during parental absences (for similar results in other settings see [39–41]). Thus, from early age, Baka children hunt birds or rodents and gather sub-spontaneous tubers, all products which typically are immediately cooked and eaten by the children themselves.

An important finding of our work relates to gendered differences of Baka children’s daily activities, a finding that dovetails with other studies both in farmer [37, 42] and hunter-gatherer [13] societies. The finding, however contrast with at least one study reporting few differences in the activities performed by girls and boys among the Aka, another hunter-gatherer group from the Congo Basin [16]. Baka children do tend to reproduce adult’s same-sex activities. Thus, as Baka women, Baka girls are more involved in children caretaking, cooking, agricultural work and fishing than Baka boys. Similarly, as Baka men, Baka boys are more often involved in hunting than Baka girls.

In addition to gendered differences in frequency of engagement in certain activities, our results also suggest that there are additional differences in the way activities are practiced. For example, girls occasionally hunt. But they only hunt little mammals using their hands, the machete or, during adolescence, unearthing game with smoke. Boys, however, not only hunt more frequently, but they also use a broader diversity of techniques, such as bow and arrows, slingshot, spear and snares. Contrarily, fishing is more frequent among girls, who typically engage in collective fishing expeditions, in which a group of girls and women elevate dams in shallow rivers and extract the water to catch the fishes with their hands. Differently, although boys also fish, they are more likely to use poles or ichtyotoxics, techniques practiced generally alone or in small groups (for a similar finding see [Díaz-Reviriego I. et al., *under review*]).

It is worth noting, however, that while some activities are clearly gender-oriented, there are not strict gender exclusions in the performance of most activities. Thus girls and boys, as women and men, occasionally perform activities most commonly performed by people from the opposite sex. The flexibility in activity performance, beyond standard gender roles, is a common, but seldom noted distinction of hunter-gatherers versus farmers [43].

The study of children’s involvement in daily activities also shows that preferred activities change as children grow up. As Aka children [11], Baka children tend to spend less time playing and more time in productive and specifically in income generating activities (i.e., agricultural wage labor or commercial hunting and gathering) as they move into adolescence.

In sum, consistent with the Whitings’ [10] predictions, the descriptive analysis of Baka children’s daily activities suggests that such activities are largely shaped by their specific cultural settings, although the sex and the age of the child are important factors that pattern children’s involvement in activities.

### Knowledge acquisition through daily activities

We devote the second part of the discussion to analyze how the frequency of performance of different activities might shape LEK acquisition and to describe how such acquisition varies according to the age and the sex of the children.

First, as mentioned, subsistence related activities are predominant during Baka childhood. Additionally, most of the activities Baka children perform through all their childhood occur in their natural environment. We argue that the performance of such activities might directly contribute to local knowledge acquisition, and more specifically to the acquisition of local ecological knowledge. For example, during their youngest childhood, boys spend a considerable amount of time hunting and girls invest time fishing, allowing them to embody hunting and fishing knowledge. Time involved in both activities decreases once they become adolescents; however as knowledge is already embodied, adolescents are able to practice these activities even if they do so more infrequently.

Thus, an important aspect to consider when discussing LEK acquisition and the performance of daily activities relates to the variation, across the lifespan, in the use of techniques and practices of different complexity. Overall, the number of practices and the complexity of tools children use during their daily activities increases with age. Take the case of hunting. From the earliest age, children play various hunting-related games, such as shooting wheels or throwing spears to easy-to-target objects and animals. Then, during middle childhood they start to use popular hunting tools, such as the , or small replicas of the common snare with cable used by adults (called ). Differently, adolescents prefer hunting with spears or the collective hunting of small mammals using smoke. The case of gathering of wild edibles is similar. Even young children gather tubers of spontaneous agricultural plants, such as  (*Ipomoea batatas),* and  (*Xanthosoma mafaffa),* which typically grow around Baka villages. Differently, adolescents gather the leaves of  (*Gnetum africanum*), an important component of the household consumption, or other forest products, such as *mbalaka* (*Pentaclethra macrophylla)*, *(Baillonella toxisperma),* or *payo (Irvingia excelsa)* which can be sold in local markets. Although gathering does not require many tools and techniques (except for some specific products such as honey and yams), effective gathering requires the acquisition of knowledge related to observation, the capacity to identify wild edibles, and the ability to navigate the landscape, abilities that also evolve across the lifespan. In sum, the analysis of children’s activities suggests that LEK acquisition seems to follow a ‘multi-stage learning model’, according to which children would first acquire basic knowledge and abilities that would allow them to progressively acquire more complex skills and knowledge [44].

One more point requires attention. Although the predominance of activities related to household maintenance might apparently underscore our argument of the importance of subsistence-related activities for LEK acquisition, we argue that the performance of these activities are key to obtain the cultural bases of adult’s livelihood and, therefore for LEK acquisition. Indeed, activities such as fetching water, collecting firewood, cooking, sharing meals, or even taking care of younger siblings, are considered by parents as key elements in Baka children’s learning process. Additionally, it is worth noticing that while conducting such household chores, especially those that take place in the forest, children also engage in other activities such as hunting birds, fishing, or gathering mushrooms. Adults clearly know that those tasks let children learn and practice on their own, alone or in groups of peers, skills that they would later need.

Our final point relates to the engagement in activities not traditionally performed by the Baka, such as schooling or listening to recorded music in bars [24]. Consistent with previous studies, both among the Baka [45, 46] and among other hunter-gatherer groups [13, 47], we found that school attendance was very limited, but existent. Thus, for the school year 2012–2013, 54 % of the schooled-aged children were registered at school. However, school attendance was low, irregular, and decreased as the school year advanced. Reasons for this low attendance do not differ from those highlighted by Kamei [46] (i.e., teachers’ low level of commitment) and largely reflect the lack of fit between the national educative system and Baka’s livelihood. Our data also show that new leisure activities, i.e., listening to recorded music, were increasingly common among Baka children. From our ethnographic experience, we know that Baka children generally listen to African popular music during the evening when they might also drink alcohol. These activities, generally very common among all the Baka, are now included in the standard use of time of adolescents and young adults, as they seem to have become the new way of socializing and even of potentially finding a partner [48].

As such activities are new in Baka repertoire, we have no way to assess how their performance might affect LEK acquisition. Studies focusing on the impact of schooling on LEK have found that schooling might have a negative impact on LEK acquisition, unless schooling is adapted to the local social-environmental context [47]. One could speculate that the case would be similar for the Baka. The work presented here, however, brings to the light that not only schooling, but also non-traditional leisure activities might impact the process of LEK acquisition. For example, activities that are mostly conducted at night, such as listening to recorded music in bars, might displace other cultural activities such as the performance of tales, songs and traditional dances, many of which begin as night falls [49]. Interestingly, none of the previous studies on the erosion of LEK have addressed the impact of children’s involvement in new leisure activities in LEK acquisition. Such neglect is worrisome, especially if-as results from our study show-children devote more time to modern forms of leisure than to schooling.

## Conclusion

Results from this work bring new elements to enhance our understanding of hunter-gatherer children’s daily life and of how those activities relate to the process of knowledge acquisition. Our results suggest that further research on LEK acquisition and children’s daily life should analyze data disaggregated by children’s sex and age, as these two characteristics seem to largely shape children’s choice of activities. Moreover, such research might be enriched by increasing the attention to understudied aspects, such as children’s experience and embodiment of basic knowledge and skills.

Playing more attention to the study of children’s daily activities can help predict changes potentially affecting small-scale societies, as for example the involvement in new leisure probably predicts less knowledge acquisition. However, longitudinal data would be needed to properly evaluate the long-term impact of activities that are new in Baka livelihood (see for example [50]). In that sense, data presented here constitutes a valid baseline for future research.
